# Aggressive Systemic Mastocytosis Presenting as Pyrexia of Unknown Origin and Sclerotic Bone Lesions: An Uncommon Presentation of a Rare Haematological Disorder

**DOI:** 10.7759/cureus.92162

**Published:** 2025-09-12

**Authors:** Umar Ismail, Syeda Ambreen Fatima

**Affiliations:** 1 Internal Medicine, Withybush General Hospital, Hywel Dda University Health Board, Pembrokeshire, GBR

**Keywords:** advanced mastocytosis, fever, fever of unknown origin (fuo), hepatosplenomegaly, malignancy of unknown primary, mastocytosis, oncology haematology, pyrexia of unknown origin (puo), rare diseases, tryptase

## Abstract

Mastocytosis is a heterogeneous disease characterised by mast cell infiltration into various organs. While cutaneous manifestations are easily recognisable, systemic disease is often difficult to diagnose. Systemic mastocytosis (SM) presenting as pyrexia of unknown origin (PUO) and sclerotic bone lesions is uncommon and adds a layer of complexity to an already challenging diagnosis, especially in the elderly where differential diagnoses are broad. We report the case of an 83-year-old man with recurrent hospital admissions due to unexplained fever, weight loss and cytopenias. Notably, the patient had no prior history of skin lesions, anaphylaxis, or other classical signs of mast cell activation. Computed tomography (CT) of chest and abdomen showed sclerotic rib lesions and hepatosplenomegaly. Extensive workup for infectious, autoimmune, and malignant diseases did not yield a diagnosis. Although a wide range of differential diagnoses were considered, serum tryptase was never tested. Eventually a bone marrow biopsy was done and showed multifocal dense mast cell infiltrates expressing CD117, CD25, and CD2 on immunohistochemistry. Serum tryptase done post bone marrow biopsy was elevated at 810 ug/L. The KIT D816V mutation was confirmed on genetic analysis. Disease was classified as aggressive SM on the basis of organ dysfunction, cytopenias and high mast cell burden. The patient responded well to midostaurin and supportive therapy, with quick resolution of fever and systemic symptoms. Tryptase levels declined steadily over a year. This case underscores the value of serum tryptase and early bone marrow examination in the assessment of PUO, especially when extensive investigations have not yielded a diagnosis. SM is an unusual and potentially under-recognised cause of PUO that should be considered early on in its differential diagnosis, especially when conventional workup is unrevealing. A high index of suspicion is required for diagnosis especially in the absence of recognisable symptoms of mast cell activation. Early diagnosis is essential for effective treatment, prognostication and to alleviate its impact on mental wellbeing. Clinicians should consider incorporating serum tryptase into investigation algorithms for PUO in patients with unexplained cytopenias, organomegaly and bone lesions.

## Introduction

Derived from CD34+/CD117+ pluripotent haematopoietic progenitor cells of the bone marrow, mast cells (MCs) are a key component of the innate immune system and are widely distributed throughout connective tissues in the skin, liver, spleen, lymphatics, gastrointestinal and respiratory tracts [1]. They mediate acute inflammation, type 1 hypersensitivity reactions and anaphylaxis through the production of vasoactive mediators, arachidonic acid metabolites and cytokines that influence the behaviour of other immune cells.

Mastocytosis is an umbrella term for a heterogeneous group of haematological disorders that have at their core the clonal expansion of MCs with distinct genetic mutations involving the stem cell factor receptor KIT (CD117). KIT is a type III transmembrane receptor tyrosine kinase expressed by approximately 1-4% of nucleated cells in normal human bone marrow, including the majority of CD34+ cells [1]. However, on maturation all haematopoietic lineages except MCs downregulate their expression [1]. Stem cell factor-KIT interactions are essential for the development of MCs, melanocytes, erythrocytes, and germ cells [2]. A somatic gain-of-function mutation in different regions of the receptor is present in >90% of patients with mastocytosis. The KIT D816V mutation is the most common, although many other mutations have now been described [1]. These mutations lead to alterations of protein structure that result in constitutive activation of the receptor independent of its ligand [2]. Activated KIT binds to intra-cellular substrates and phosphorylates them, switching on multiple signalling pathways by interacting with enzymes and adaptor proteins, the end result of which is enhanced differentiation, survival and activation of MCs [2].

The World Health Organization (WHO) classifies mastocytosis into cutaneous mastocytosis (CM), systemic mastocytosis (SM), and MC sarcoma (MCS). SM is further sub-classified into indolent SM (ISM), smouldering SM (SSM), aggressive SM (ASM), SM with an associated clonal hematological non-MC lineage disease (SM-AHNMD), and MC leukaemia (MCL) [3]. ASM, SM-AHNMD and MCL are together referred to as advanced SM and carry a poor prognosis [3]. SM as a group makes up only about 1.5% of all myeloid tumours, but advanced SM is even more rare. A Danish nationwide cohort study for the period between 1997 and 2010 reported a prevalence of 9.59 per 100,000 inhabitants for all subtypes of SM [4]. The distribution of ISM and advanced SM provided by the Danish study and an Italian multicentre study reported a relatively high frequency of ISM (82-89%) and low frequency of advanced SM (7%), which consists of SM-AHNMD (4-5%), ASM (2-6%), and MCL (0.2-1%). Eleven percent of cases in the Danish study were reported to be of an unknown subtype [1,4]. Their ubiquitous distribution and the large repertoire of mediators they produce make MC disorders challenging to diagnose in clinical practice. The clinical manifestations are related to the release of mediators such as histamine, tryptase, prostaglandins, leukotrienes, and cytokines. These mediator-related symptoms can be mild, moderate, severe or life-threatening depending on genetic variables and comorbidities. These symptoms include pruritus, flushing, blistering, abdominal pain, diarrhoea and, in severe cases, hypotensive episodes and anaphylaxis [1]. Additionally, destructive infiltration of MCs in advanced forms of the disease can cause symptoms related to end-organ dysfunction including malabsorption and weight loss, osteolysis and/or osteoporosis with pathologic fractures, hypersplenism, hepatomegaly with impairment of liver function (often with ascites), and significant cytopenias [5].

Although generally not an easy diagnosis to make due to low physician awareness, especially in primary care, mastocytosis is relatively easier to suspect in patients with urticaria pigmentosa (UP), unexplained or severe insect-induced anaphylaxis, or symptoms of mast cell degranulation without a true allergy [6]. However, while unexplained haematological abnormalities like cytopenias or sclerotic bone lesions may rarely be the initial presentation of the disease, they don't always trigger clinical suspicion. These are more likely to trigger suspicion for common conditions like bone marrow infiltration from solid tumours such as prostate cancer, especially in an elderly male population. Patients above 80 years of age present a particularly challenging diagnostic prospect due to both the rarity of the disease in this age group, the frequency of atypical symptoms and clinical presentations often complicated by other comorbid conditions that may readily explain the symptoms. A Swedish population-based study (2001-2018) tracked 4,438 SM diagnoses among adults. Only about 4.5% (n = 91) occurred in individuals aged 80-89 years, and 0.6% (n = 12) in those aged 90 and above [7]. Given that ASM makes up roughly 2-6% of all SM cases, the incidence in individuals aged 80 years and above is correspondingly minimal, likely well under 0.1 per 100,000 per year. In the case presentation below, we explore the convoluted journey of one such elderly patient, from first contact with primary care to final diagnosis, highlighting the challenges faced and possible areas for improvement. Patient provided written informed consent.

## Case presentation

We present an 83-year-old man with benign prostatic hyperplasia who first presented to primary care with shortness of breath. He denied any weight loss or rectal bleeding. General physical examination including digital rectal exam was normal. Routine investigations such as full blood count (FBC), haematinics and chest x-ray (CXR) were done. Test results showed mild anaemia with haemoglobin of 120g/L (normal 130-180g/L) and low vitamin B12 at 100ng/L (normal 185-914ng/L). CXR was normal. Additional investigations including prostate-specific antigen (PSA) and serum immunoglobulins were normal. Supplementation was started for B12 and an urgent referral was made to colorectal surgeons for additional evaluation to exclude bowel malignancy. Referral was however declined as faecal immunochemical test (FIT) was normal.

**Figure 1:**
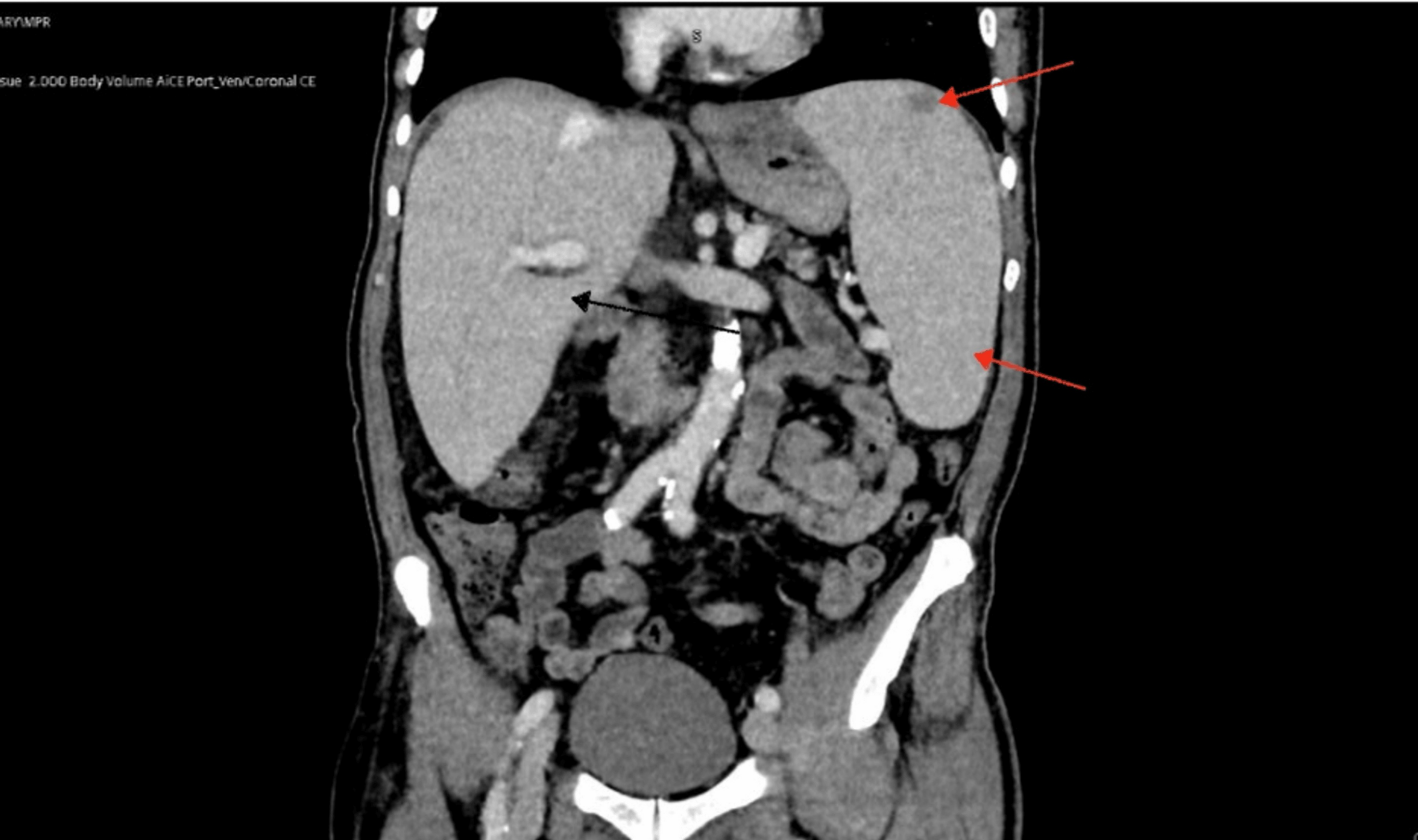
Computed tomogram thorax abdomen and pelvis (CT TAP) showing hepatomegaly (black arrow) splenomegaly (bottom red arrow) and hypodense lesion in the dome of the spleen (top red arrow)

Patient was admitted to hospital in the following week due to worsening shortness of breath, fatigue and weight loss. Vital signs and systemic examinations were normal; electrocardiograph (ECG) was also normal and blood tests were stable. Computed tomography pulmonary angiogram (CTPA) was requested to exclude pulmonary embolism (PE). Although PE was ruled out, CTPA showed splenomegaly and sclerotic rib lesions concerning for metastatic deposits. Mild bronchial wall thickening suggestive of chronic airway inflammation was also noted. This was followed up with a computed tomography of thorax, abdomen and pelvis (CT TAP), which confirmed multiple sclerotic rib lesions, hepatosplenomegaly and mild ascites and bilateral pleural effusions (Figure 1). A note was also made of a hypodense lesion within the dome of the spleen. Patient improved on admission and was discharged home. A referral was made to the malignancy of unknown origin multidisciplinary team (MUO MDT) for further evaluation. A bone scan was also arranged to further characterise CT findings.

**Figure 2:**
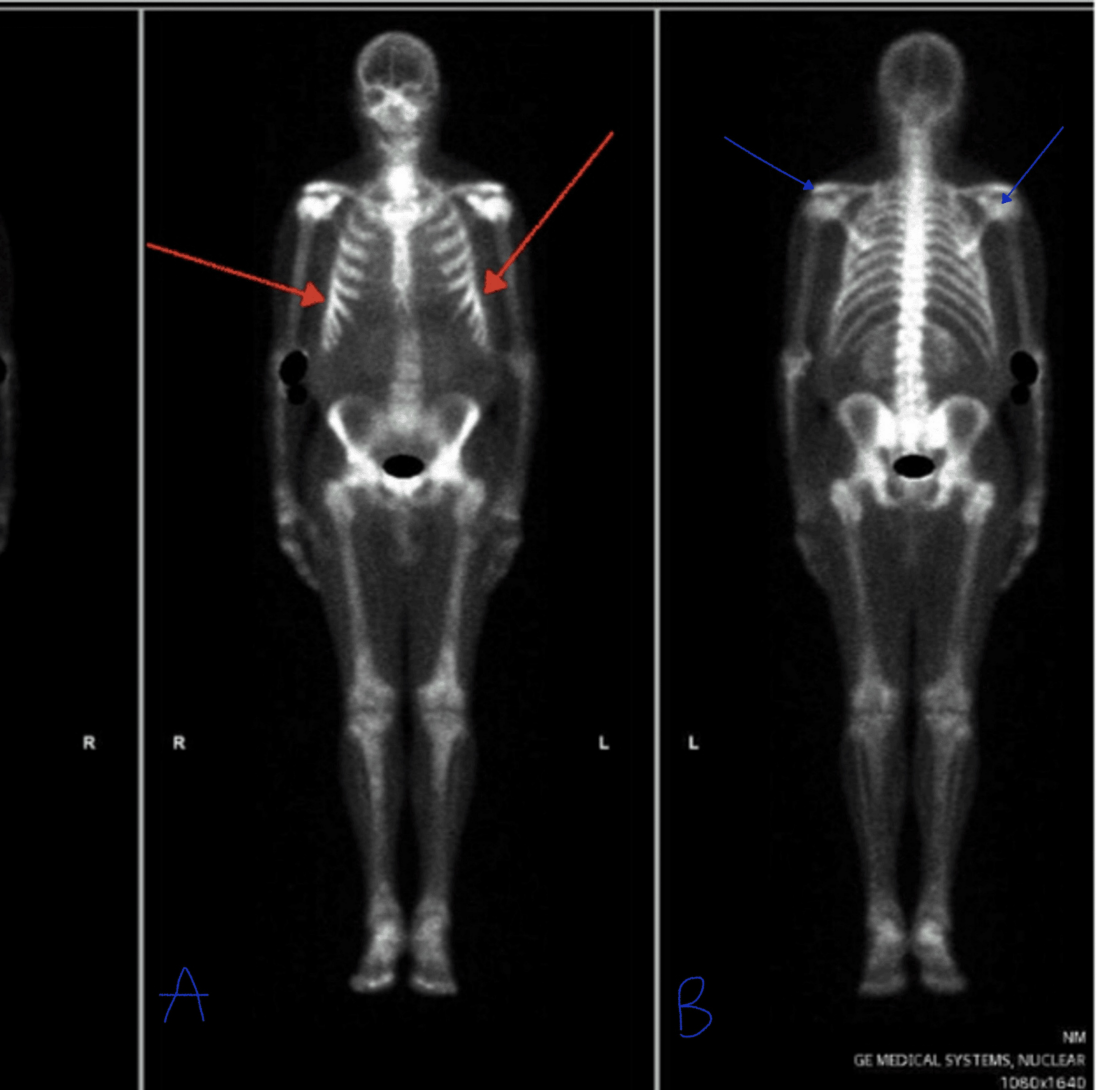
Nuclear medicine (NM) bone scan showing increase activity in anterior ribs (red arrows in image A). Increased activity in the acromioclavicular and gleno-humeral joints is due to degenerative disease (blue arrows in image B)

However, the patient re-presented to hospital less than 24 hours post discharge with generalised aches, cough, rigors and fever. He reported worsening shortness of breath and a near-collapse episode. On examination he was found to be febrile, tachypneic, tachycardic and mildly confused. Skin and other systemic examinations were normal; no wheeze was detected on chest examination. Routine investigations (FBC, metabolic profile, C-reactive protein) were done and a diagnosis of chest infection was made for which he was started on IV antibiotics. He continued to improve during the admission and was subsequently discharged on oral antibiotics. The patient was discussed in the MUO MDT soon after discharge, and a clinic review was planned while awaiting results of pending investigations (bone scan, testicular ultrasound and MUO tumour markers). A bone scan was done and showed foci of subtle activity in the ribs bilaterally (Figure 2), features suspicious for metastatic rib deposits. The testicular ultrasound returned normal.

Patient was admitted to hospital again a week later due to worsening shortness of breath and high fever. Clinical examination and vitals were unremarkable; CXR was normal. Blood tests were done, which showed deranged liver enzymes (raised alkaline phosphatase and gamma-glutamyl transpeptidase), anaemia and thrombocytopenia. Folate and transferrin saturations were low, and prothrombin time was prolonged. Antibiotics were given in hospital and the patient remained fever-free until subsequent discharge home. MUO MDT felt that on balance, sclerotic rib lesions were benign. Patient was kept under surveillance due to the ongoing hepatosplenomegaly, deranged liver enzymes and cytopenias. Haematology consult and liver biopsy were requested. Haematologist recommended repeating FBC with peripheral blood film, parietal cell and intrinsic factor autoantibody tests, thyroid function tests and coeliac screen. Vitamin K and fresh frozen plasma were recommended prior to liver biopsy to normalise coagulation. Folate and iron replacement were also suggested.

**Figure 3:**
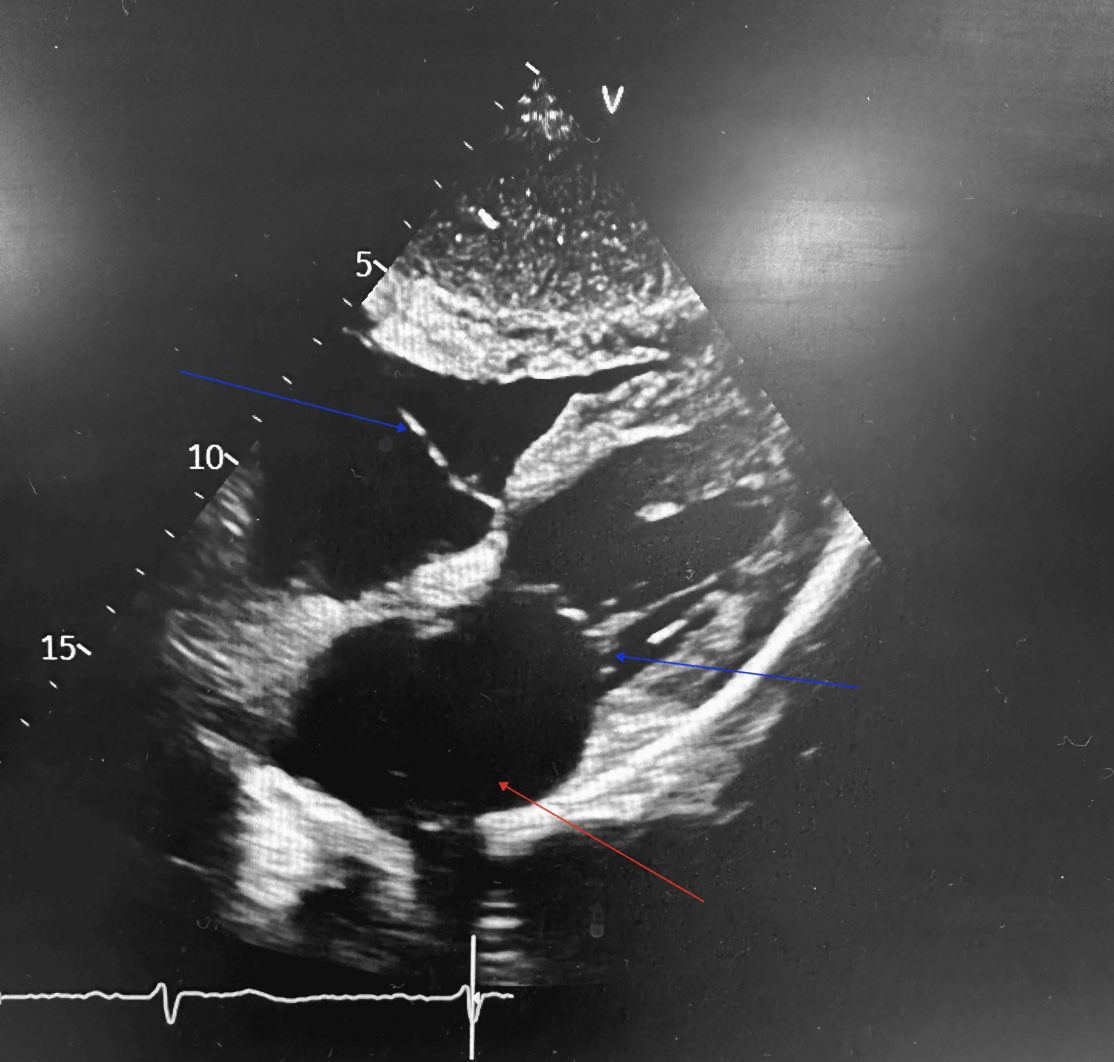
Echocardiogram showing mild left atrial dilatation (red arrow) and clear valves with no vegetations (blue arrows)

Patient was again admitted with the same symptoms less than two weeks post discharge. Investigations showed mildly raised inflammatory markers (CRP and ESR) and worsening anaemia (Table 1). Vasculitis screen and blood cultures were negative. Differential diagnoses such as prostate cancer, plasma cell dyscrasia and lymphomas were entertained, and tumour markers such as alpha-fetoprotein, PSA, lactate dehydrogenase, Bence-Jones proteins and free light chains were requested and returned negative. Raised alkaline phosphatase and gamma-glutamyl transpeptidase were noted with otherwise stable liver function as shown in Table 1. The rib lesions were considered unsuitable for biopsy as they were inaccessible. Liver biopsy was completed and did not show any histological evidence of malignancy, infection or inflammation. There was no cirrhosis seen to account for the splenomegaly. Patient continued to have fever overnight but was well enough during the day. Transthoracic echocardiogram (ECHO) done to screen for infective endocarditis did not show any valve vegetation but did show mild left ventricular systolic impairment with ejection fraction of 49% and mild left atrial dilatation (Figure 3). Trans-oesophageal ECHO was discussed with cardiologists. In view of the pyrexia of unknown origin (PUO), a positron emission tomography (PET CT) scan was also arranged. Patient was discharged home and was to be seen in the MUO clinic the following week.

**Table 1:** Summary of relevant lab results MCV: Mean corpuscular volume, CRP: C-reactive protein, K: Potassium, ALP: Alkaline phosphatase, GGT: Gamma glutamyl transpeptidase, AST: Aspartate transaminase, ALT: Alanine transaminase, LDH: Lactate dehydrogenase, RF: Rheumatoid factor, ANCA: Antineutrophil cytoplasmic antibody, ANA: Antinuclear antibody, ACE: Angiotensin converting enzyme, ELISA: Enzyme linked immunosorbent assay, MTB: Mycobacterium tuberculosis, EBV: Epstein-Barr virus, PCR: Polymerase chain reaction, INR: International normalised ratio, NA: Not applicable

Labs	Value	Reference range
Complete blood count and Metabolic Profile		
White cells	6.6	4.5- 11 k/uL
Neutrophils	4.7	1.8-7.5 k/uL
Haemoglobin	85	130-180 g/L
Platelets	90	150- 400 k/uL
MCV	99	80-100 fL
Eosinophils	0.9	0.1-0.4 k/uL
Lymphocytes	0.6	1- 4 k/uL
Creatinine	115	58-110 umo/L
Urea	7.8	2.5-7.8 mmol/L
CRP	25	<5 mg/L
K	4.4	3.5-5.5 mmol/L
ALP	347	30-130 U/L
GGT	314	5-40 U/L
Ferritin	788	15-300 ug/L
Bilirubin	11	< 21 umol/L
ALT and AST	<50	<50 U/L
Albumin	41	35-55 g/L
Vitamin D	70	>50 nmol/L
LDH	122	140-280 U/L
Autoimmune screen		
RF	<10	<10 IU/mL
ANA	Negative	NA
ANCA	negative	NA
ESR	61	5 mm/hr
ACE	26	20-70 U/L
Microbiology		
Q-fever 1 and 2	Negative	NA
Bartonella screen	Negative	NA
Coxiella burnetti ELISA	IgG & IgM Negative	NA
Quantiferon TB gold	Negative	NA
Brucella serology	Negative	NA
Borrelia screen	Negative	NA
MTB culture	Negative	NA
EBV PCR	Negative	NA
Toxoplasma serology (IgM)	Negative	NA
Syphilis serology	Negative	NA
Blood cultures	Negative	NA
Hepatitis B&C	Negative	NA
Miscellaneous		
Tryptase	810	0-12.9 ug/L
INR	1.4	0.8-1.2

**Figure 4:**
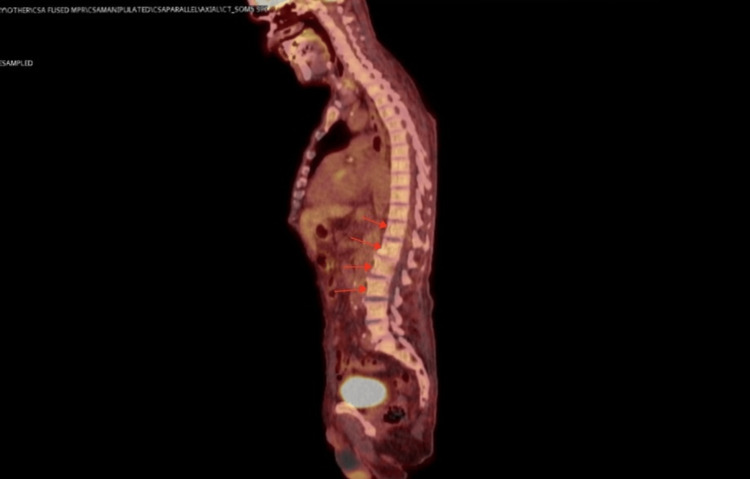
Fluoro-deoxyglucose positron emission tomography (FDG-PET) scan showing sclerotic vertebral body lesions without hyper-metabolic activity (red arrows)

**Figure 5:**
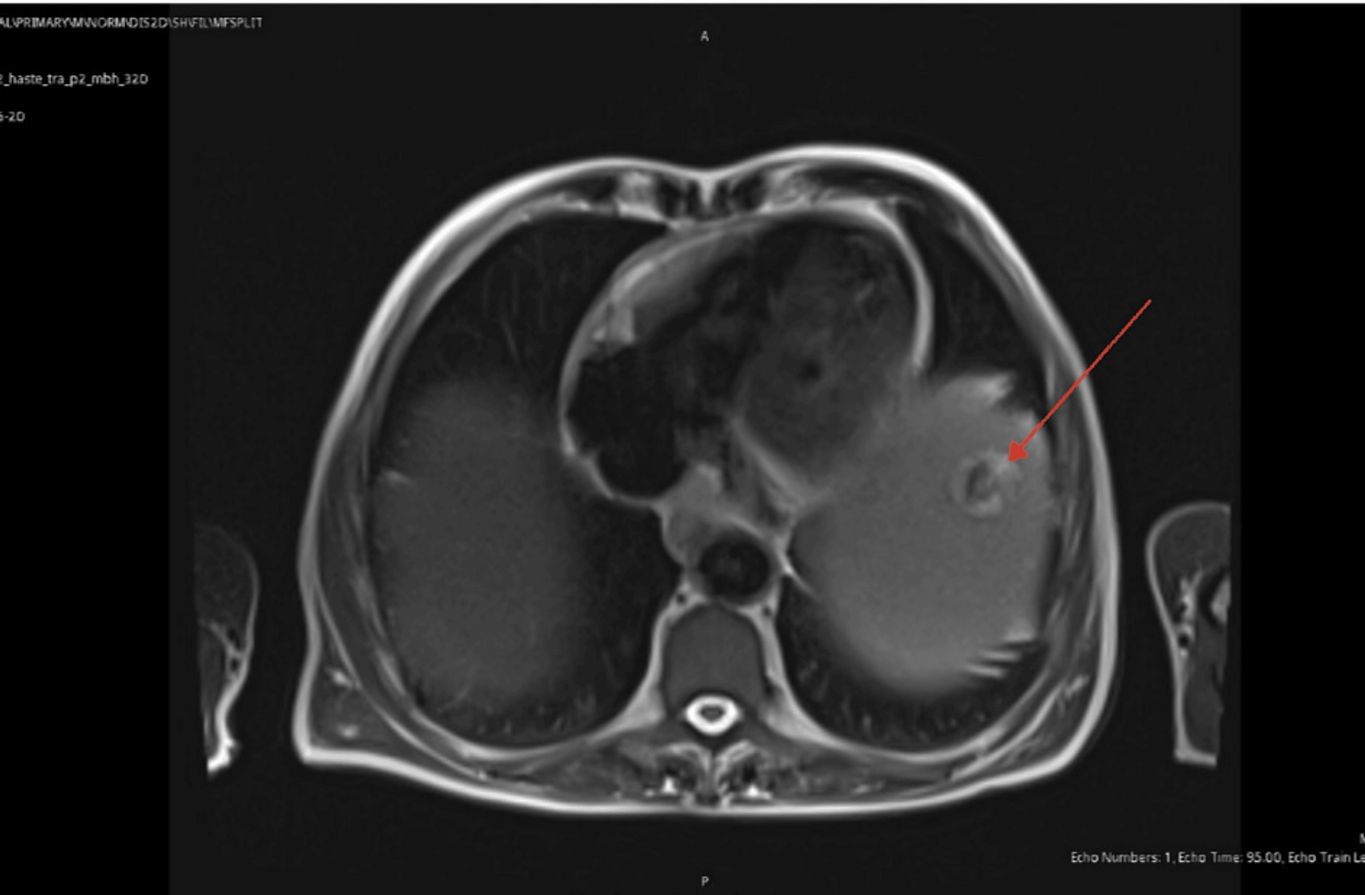
Magnetic resonance imaging (MRI) of liver and spleen showing largest of three splenic lesions (red arrow), other two lesions are not shown

PET-CT scan, Quantiferon test for tuberculosis and bone marrow biopsy were requested. Some concerns were expressed over possible culture-negative infective endocarditis, but no clinical signs were present. Microbiology workup for culture-negative infective endocarditis including screens for Q fever, Bartonella and Borrelia came back negative; blood-borne viruses screen also came back negative (Table 1). PET CT scan showed no site of inflammation or infection and no evidence of vasculitis (Figure 4). A small hypermetabolic focus corresponding to a left thyroid cyst was noted and a suggestion was made for thyroid ultrasound, which showed indeterminate nodule. Magnetic resonance imaging (MRI) of liver and spleen was suggested and showed hepatosplenomegaly with three focal splenic lesions, the largest measuring 2.5 cm in diameter (Figure 5).

**Figure 6:**
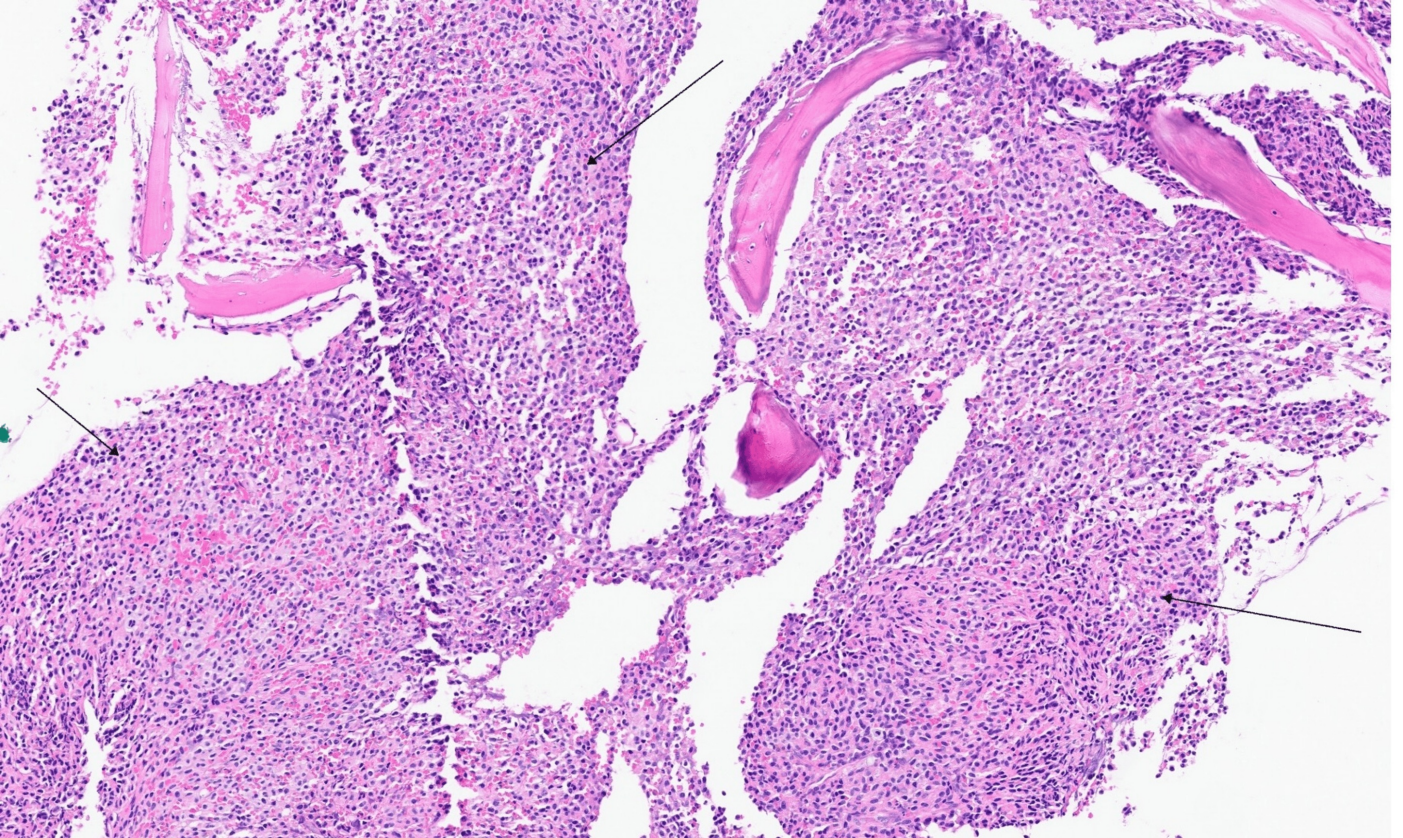
H&E stain showing infiltrates of spindle-shaped cells with clear eosinophilic cytoplasms and elongated nuclei in keeping with neoplastic mast cells (black arrows) H&E: Hematoxylin and Eosin

**Figure 7:**
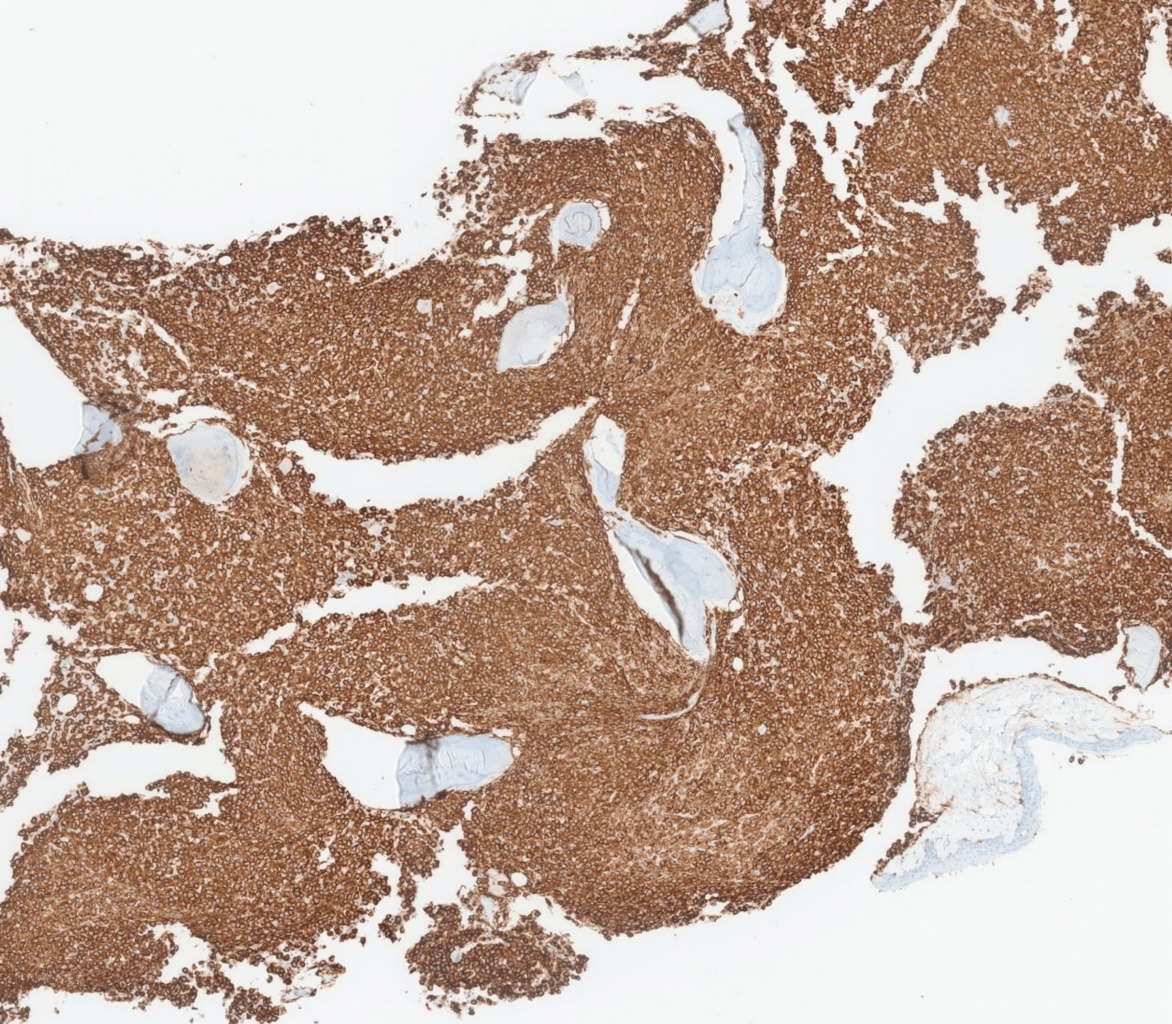
CD117 immunohistochemical stain showing widespread infiltration of bone marrow by CD117+ mast cells (staining brown)

**Figure 8:**
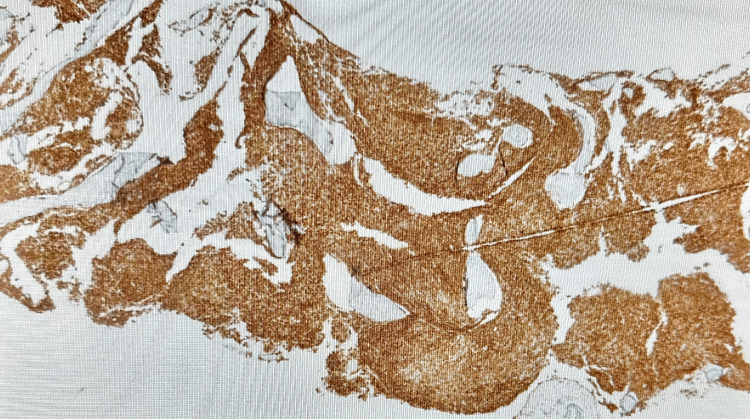
Immunohistochemical stain showing CD25+ mast cell infiltrates in the bone marrow (staining brown)

Patient was admitted to hospital for the fourth time, roughly three months since first presentation to primary care, again with fever, shortness of breath and no other symptoms. Systemic examinations were stable; blood tests were also stable in comparison to recent findings. Bone marrow trephine biopsy was subsequently performed and showed massively hypercellular bone marrow packed with infiltrates of small spindle-shaped cells with abundant pale cytoplasm and regular, ovoid hyperchromatic nuclei (Figure 6). Residual haematopoietic tissue was difficult to discern within the infiltrates. The cell populations replacing the marrow were CD2+, CD117+ (Figure 7) and CD25+ (Figure 8), in keeping with neoplastic MCs. The appearances were of a bone marrow packed with MC infiltrates, in keeping with SM. Biopsy did not show any evidence of associated non-MC lineage haematologic neoplasm. The cKIT D816V variant was detected at a level of 11.5% from bone marrow tissue using droplet digital polymerase chain reaction (ddPCR). Subsequent testing for MC tryptase done after biopsy came back elevated at 810 ug/L.

The patient met the major criterion for SM: multifocal clustering of mast cells (>15 cells per cluster) in an extracutaneous organ and all minor WHO criteria. He also met the specifications for aggressive SM due to bone marrow dysfunction with cytopenias and hepatosplenomegaly with ascites. 

Patient started treatment with midostaurin 50 mg twice daily and was transfused with 1 unit of red cells as haemoglobin was low. Patient initially felt groggy and had bouts of emesis after starting midostaurin. These are recognised common side effects of midostaurin that were managed easily with symptomatic treatment. He quickly improved with antiemetics, feeling better with complete resolution of vomiting and pyrexia. Breathing also improved. Other more serious toxicities including interstitial lung disease, cardiac toxicity (arrhythmias), thrombocytopenia and hepatotoxicity, which would have necessitated dose adjustment or discontinuation, were not immediately observed. 

Patient's family had some concerns over plans for discharge home given multiple hospital admissions but were reassured after speaking to senior physicians. He was discharged home successfully and had no subsequent hospital admissions. Tryptase levels declined by above 50% one month after starting midostaurin. Patient remained well one year after starting treatment and was extremely grateful to the team for the care he received as he felt his quality of life had improved. He reported being able to take daily walks (something he enjoys and could not do previously before starting treatment). The serial response in cell counts and biochemical markers after starting treatment with midostaurin is shown in Table 2 below.

**Table 2:** Serial response in cell counts and biomarkers BM: Bone marrow, SM: Systemic mastocytosis, QoL: Quality of life, ALP: Alkaline phosphatase, GGT: Gamma glutamyl transpeptidase

Date	Time from start of midostaurin	Serum tryptase (µg/L)	Haemoglobin (g/L)	Platelets (×10⁹/L)	ALP (U/L)	GGT (U/L)	Key clinical notes
Jul 2024	Baseline (Day 0)	810	85	90	347	314	BM biopsy confirmed SM; KIT D816V mutation burden 11.5%; midostaurin 50 mg twice daily started
Aug 2024	~1 month	319	95	73	197	222	Symptoms resolved; no further fevers; improved QoL
Oct 2024	~3 months	157	120	111	100	62	Symptom-free
Jan 2025	~6 months	172	120	117	50	60	Symptom-free
Jul 2025	~12 months	162	120	107	50	40	Sustained remission; persistent thrombocytopenia; dose maintained at 50 mg twice daily

## Discussion

The case presentation highlights the difficulties encountered by patients presenting to healthcare services with rare diseases, especially when symptoms are non-classical or evolving. Mastocytosis is more commonly cutaneous and presents with typical skin changes (UP) in a younger population. Systemic disease on the other hand has a broad clinical presentation linked to both systemic symptoms of MC activation and their infiltration into organs leading to dysfunction. This protean presentation can lead to diagnostic delays. A retrospective study from the Netherlands reported an average time to diagnosis of 8.1 years [8]. Another retrospective cohort study involving 116 patients in a large U.S. healthcare system found a mean delay of 58.3 months from symptom onset to confirmed SM diagnosis using WHO diagnostic criteria [9]. Our patient's time to diagnosis from first presentation to primary care was notably below the average reported in both studies (three months). The patient's timeline is summarised in Table 3 below.

**Table 3:** Timeline: First contact with primary care to diagnosis, commencement of treatment and subsequent follow-up FBC: Full blood count, LFT: Liver function test, FIT: Faecal immunochemical test, CTPA: Computed tomogram pulmonary angiography, CT TAP: Computed tomogram thorax abdomen and pelvis, MUO-MDT: Malignancy of unknown origin multidisciplinary team, MRI: Magnetic resonance imaging, CRP: C-reactive protein, ESR: Erythrocyte sedimentation rate, PET-CT: Positron emission tomography, ECHO: Echocardiograph, EF: Ejection fraction, PUO: Pyrexia of unknown origin, FFP: Fresh frozen plasma, ASM: Aggressive systemic mastocytosis

Timeline	Event	Key Findings	Relevant Investigations	Interventions	Notes
15^th^ March 2024	First contact with primary care: Shortness of breath.	Anaemia with Hb of 120g/L and low vitamin B12 100ng/L	FBC; Metabolic profile; Vitamin B12; Iron levels; FIT	B12 supplementation	Referral to colorectal surgeons for further anaemia evaluation
16^th^ to 22^nd^April 2024	First hospital admission: Fever, shortness of breath and weight loss	Cytopenias, hepatosplenomegaly, splenic lesions, mild ascites and pleural effusion Sclerotic bone lesions	FBC; LFTs; blood cultures; CTPA and CT TAP	Empirical IV antibiotics	Discharged after improvement; MUO MDT referral
25^th^ April to 1^st^ May 2024	Second hospital admission: Recurrent fever	Hepatosplenomegaly, worsening anaemia/thrombocytopenia, deranged LFTs. Subtle increase in activity in ribs on NM bone scan	NM bone scan; Testicular ultrasound (normal); LFTs; FBCs, Haematinics (folate and iron levels)	Supportive care, folate and iron replacement, Haematology consult; Liver biopsy planned	Persistent symptoms; Diagnosis still unclear. Discharged
9^th^ to 20^th^May 2024	Third hospital admission: Persistent fever	Worsening anaemia, hepatosplenomegaly and splenic lesions on MRI; Normal histology of liver biopsy specimen; No hypermetabolic bone activity on PET-CT	MRI liver; CRP; ESR; Liver biopsy; Transthoracic ECHO (EF 49%); PET-CT scan; Microbiology work up for culture negative endocarditis	Symptom control, vitamin K and FFP prior to liver biopsy	Bone marrow biopsy planned
17^th^ June to 11^th^ July 2024	Fourth admission: Recurrent fever and shortness of breath	Bone marrow biopsy: Dense mast cell infiltrates, CD117/CD25/CD2 positive; Serum Tryptase: 810ug/L; KIT D816V mutation detected	Extensive investigations for PUO (microbiology, blood panel, autoimmune screen); Bone marrow biopsy; Immunohistochemistry; Flow cytometry; Serum tryptase; KIT mutation testing	Treatment plan formulated Supportive care midostaurin 50 mg twice daily	Diagnosis confirmed; Major WHO criteria met for systemic mastocytosis; WHO criteria fulfilled for ASM
11 August 2024	One-month follow-up	Symptomatic improvement; >50% fall in serum tryptase	FBC; LFTs; Serum tryptase	Continued midostaurin	No further fevers; Improved quality of life
July 2025	Annual follow-up	Sustained remission; Stable blood cell counts, Persistent thrombocytopenia, stable tryptase	FBC, serum tryptase	Midostaurin ongoing	Symptoms free; No re-admission to hospital in one year; midostaurin maintained at same dose due to persistent thrombocytopenia

The patient first presented to primary care with what was believed to be symptomatic anaemia. Efforts to get him seen by colorectal surgeons were rightly rebuffed. Although colorectal cancer is a common cause of anaemia in this age group, he did not have any of its features and screening test was negative. Self-presentation to hospital ensured he was picked up by physicians and referred to the right pathway. However, that was not the end of his ordeal. Patient's symptoms evolved rapidly into what was effectively PUO with its corresponding broad list of differential diagnoses that required systematic elimination. The combination of PUO, cytopenias and sclerotic bone lesions without other classical features of MC activation made this case very unusual for SM. However, there were hints along the way that if picked up could have shortened the time to diagnosis. This highlights the importance of pattern recognition in clinical diagnostics. Taken in isolation, the patient's clinical symptoms, signs and investigation results appear complex. However, a pattern emerges once they are considered side by side. Haematologic abnormalities are common in SM, anaemia affects roughly 30-50% of patients, while thrombocytopenia and leukopenia occur in about 15-20%; eosinophilia may be as high as 40% [10]. Cytopenias and organ dysfunction are more likely to be the presenting features in elderly patients with advanced SM. The patient's recurrent febrile episodes were likely a result of periodic MC activation and mediator release. MC mediators like tumour necrosis factor alpha, interleukin 1 and 6, and histamine can cause fever. Prostaglandin E2, another mediator released by MCs, is a well-known pyrogen through its effect on the hypothalamic temperature control mechanisms. 

Notably, while the patient initially presented with anaemia, he went on to quickly develop both thrombocytopenia and marked eosinophilia over the course of two months. These findings, in the context of other organ infiltration (hepatosplenomegaly, sclerotic rib lesions), should raise SM to the top of the differential diagnoses list. Interestingly, serum tryptase was not considered during initial consultation with haematologist even though it is a readily available test that can be requested by both haematologists and physicians in our centre. This suggests that the threshold for suspicion is high for mastocytosis, even among specialists. However, it's important to remember that clinical reasoning is complex, and delays in diagnosis result from both systemic and individual factors [11]. Individual clinician factors include reliance on heuristics and availability bias, while systemic factors include non-availability of structured diagnostic algorithms and decision support tools for complex cases. While some conditions are easily identifiable, others like SM present ambiguously and lead to uncertainty. In such cases an intuitive decision-making approach may not be suitable, and a more analytical approach may yield better decisions. This dual approach to clinical reasoning was highlighted by Croskerry [11]. Although sensitivity is modest (65%), serum tryptase is one of the most important laboratory tools for evaluation of MC disorders [12]. It's both relatively available and non-invasive. The caveat is that a significant subset of patients may have normal tryptase. Bone marrow biopsy with histopathological, immunohistochemical, and molecular genetic features remains the most common and definitive method for diagnosing SM [12].

Diagnosis and classification of SM are based on the 2016 WHO criteria. The diagnostic criteria consist of one major and four minor criteria, where one major and one minor or three minor criteria are required to secure diagnosis. The major criterion is multifocal clustering of mast cells (>15 cells per cluster) in an extracutaneous organ, typically the bone marrow. The minor criteria include: (1) basal serum tryptase of ≥20 ng/mL; (2) abnormal or spindle-shaped mast cell morphology in >25% of mast cells in an infiltrate; (3) aberrant expression on mast cells of the markers CD2 or CD25 or (4) presence of the D816V mutation in KIT [12]. Classification on the other hand is based on the presence or absence of WHO "B" or "C" findings. B findings refer to high MCs burden on BM biopsy (>20% infiltration), serum tryptase >200 ng/ml, signs of dysplasia or myeloproliferation with normal/slightly abnormal blood counts, mild hepatosplenomegaly, and/or lymphadenopathy. C findings refer to bone marrow dysfunction with cytopenias, hepatosplenomegaly with ascites, portal hypertension, hypersplenism, bone lesions (osteolytic) with pathological fractures, and gastrointestinal MCs infiltration leading to malabsorption and weight loss [12]. Our patient met the diagnostic criteria for SM and also met the class specifications for aggressive SM due to bone marrow dysfunction with cytopenias and hepatosplenomegaly with ascites. Classification is important both for determining the choice of treatment and for prognostication [13]. Aggressive SM carries a poor prognosis, with a median overall survival of 5.7 years based on data from the European Competence Network on Mastocytosis (ECNM) registry [14]. 

All patients with SM should be aware of potential triggers of symptomatic episodes. Hypersensitivity reactions (HR) are mainly triggered by Hymenoptera venoms in patients with CM and ISM, and by drugs in patients with advanced SM. Tryptase levels <90 ng/mL, MC bone marrow infiltration <15%, and WHO category ISM are predictors of HR. New HRs occur in 4.8% of all patients within four years [15]. Consideration should be given to provision of medical alert bracelets and epinephrine pens to use in case of anaphylaxis. Psychological well-being should also be assessed given both symptoms and delay in diagnosis can impact mental well-being [16].

The backbone of treatment for both ISM and SSM is symptom management, and H1 and H2 antihistamines remain the mainstay of symptom control. For aggressive and leukaemic forms of SM, cytoreductive treatment is indicated [17]. With the advent of KIT‐targeted tyrosine kinase inhibitors (TKIs), the use of drugs like cladribine has declined. The multikinase inhibitor midostaurin is in widespread use for patients with advanced SM. In this population it demonstrated a 60% overall response rate and good tolerability with few reported adverse events apart from hematological toxicity, nausea, vomiting, and diarrhea [17]. Our patient received midostaurin, with significant improvement of liver function and body weight; a substantial reduction in mediator‐related symptoms was also observed, with complete resolution of fever and shortness of breath, significantly improving the patient's quality of life. Although the patient remained well after one year of treatment, long-term surveillance is ongoing given the risk of relapse and disease progression in ASM. Patient continues to have monitoring with serum tryptase levels and FBCs as part of follow-up with haematologists. Persistent thrombocytopenia prevented the up-titration of midostaurin dosage beyond 50 mg twice daily. No further bone marrow examination or imaging is planned as part of follow-up, given the patient's age and frailty.

Although not applicable to our patient, other KIT-targeted therapies are also in use for SM. In June 2021, the US Food and Drug Administration (FDA) approved avapritinib for the treatment of SM. Avapritinib is a newer multi-kinase inhibitor with highly selective and potent activity against mutated c-KIT and platelet-derived growth factor receptor A mutants. The main side effects of avapritinib are edema, diarrhea, nausea, and fatigue. It is not recommended for treatment of patients with advanced SM and platelet counts below 50 × 10 k/uL due to the risk of intracranial haemorrhage [16,18,19]. 

This report has some limitations; it describes a single patient, which inherently limits the generalisability of the observations. While the temporal association between midostaurin initiation and clinical improvement is compelling, causality cannot be definitively established. Additionally, the discordance between sclerotic bone lesions on CT and the absence of FDG uptake on PET-CT was not explored in depth. This phenomenon has been described in systemic mastocytosis, where dense osteosclerosis may not correlate with increased metabolic activity, but further dedicated studies are required [20]. Finally, although cKIT D816V testing was performed using ddPCR, alternative platforms such as next-generation sequencing may offer different sensitivity thresholds, and cross-platform comparability was not assessed in this case.

## Conclusions

Despite significant progress in understanding its immunological mechanisms and pathophysiology, SM remains a complex disorder that's challenging to diagnose, especially in those without skin involvement or familiar symptoms of MC activation. Physician threshold for suspicion remains high, which along with the non-specific nature of symptoms results in significant delays in diagnosis. Physician education through organised webinars and supervised learning activities can help improve awareness. A low threshold for work-up should be encouraged. Clinicians should consider incorporating early serum tryptase and bone marrow examination into the work-up for PUO in those with unexplained cytopenias and organomegaly. This of course requires cost-benefit analysis and validation in large prospective studies.
